# A hepatic enigma: Pediatric presentation of primary biliary cholangitis

**DOI:** 10.1002/jpr3.70210

**Published:** 2026-07-27

**Authors:** Sindhura Kasturi, Rachel Schenker, Nick Shillingford, George Yanni

**Affiliations:** ^1^ Department of Pediatric Gastroenterology, Hepatology and Nutrition Children's Hospital Los Angeles Los Angeles California USA; ^2^ Department of Pediatric Gastroenterology, Hepatology and Nutrition, Mattel Children Hospital University of California Los Angeles California USA; ^3^ Department of Pathology Children's Hospital Los Angeles Los Angeles California USA

**Keywords:** cholestasis, elevated liver enzymes, liver failure

## Abstract

Primary biliary cholangitis (PBC) is a chronic autoimmune condition characterized by destruction of intrahepatic bile ducts, leading to fibrosis and cirrhosis of the liver. It is an extremely rare pediatric disease with very few pediatric cases reported to date. Here, we report the case of a 14‐year‐old female who presented with elevated liver enzymes and was subsequently diagnosed with PBC. Our case raises awareness of the possibility of PBC in children.

## 1 INTRODUCTION

Primary biliary cholangitis (PBC) is a chronic autoimmune condition characterized by destruction of intrahepatic bile ducts, leading to fibrosis and cirrhosis of the liver. Anti‐mitochondrial antibody (AMA) is highly specific for PBC, occurring in almost 90%–95% of the cases.^1^ PBC is commonly diagnosed in middle‐aged females between 30 and 60 years of age. It is an extremely rare pediatric disease with very few pediatric cases reported to date.^2^, ^3^, ^4^, ^5^ Here, we report the case of a 14‐year‐old female who presented with elevated liver enzymes and was subsequently diagnosed with PBC. This case adds to the limited existing literature and highlights the need for awareness of PBC as a cause of chronic liver disease in children.

## 2 CASE REPORT

A 14‐year‐old female was referred to the Gastroenterology clinic for evaluation of abnormal liver labs. She had a history of neutropenia, positive antinuclear antibody (ANA), and thrombocytopenia and was following with rheumatology. She had no definitive rheumatological diagnosis, though she had a working diagnosis of an evolving connective tissue disorder. She endorsed mild abdominal pain, which improved with laxative use. She denied pruritus or fatigue. There was no reported family history of liver or autoimmune disorders.

Previous lab work showed fluctuating transaminases with aspartate aminotransferase (AST) ranging from 33 to 233 U/L (normal 0–34 U/L) and alanine aminotransferase (ALT) ranging from 42 to 436 U/L (normal 0–55 U/L). She had normal bilirubin, alkaline phosphatase (Alk Phos), and International Normalized Ratio (INR) with slightly elevated gamma‐glutamyl transferase (GGT) to 82 U/L (normal 5–36 U/L). The AMA was markedly elevated to 127 (normal 0–24.9 units). She was also noted to have elevated immunoglobulin levels, including an immunoglobulin G (IgG) of 2200 (upper limit of normal 1200) and an immunoglobulin M (IgM) of 341 (upper limit of normal 330).

In the gastroenterology clinic, further lab work and imaging were performed to explore the causes of the elevated liver enzymes. Labs were reassuring, including normal hepatitis A, B, and C workup, anti‐liver kidney microsomal antibody, anti‐smooth muscle antibody, alpha_1_‐antitrypsin (A1AT) phenotype, celiac panel, ceruloplasmin level, and thyroid function test. However, AMA remained elevated at 136 units. Ultrasound revealed hepatomegaly with steatosis and mild splenomegaly without abnormal Doppler activity.

**Figure 1:**
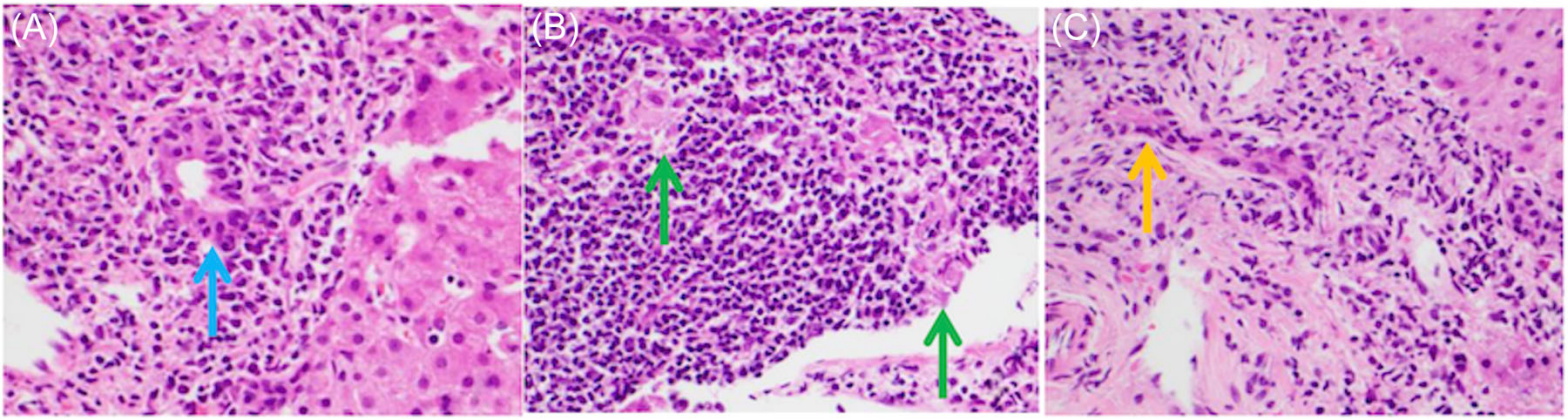
(A) Photomicrograph from the initial biopsy shows prominent portal inflammation with a predominance of lymphocytes and plasma cells without significant interface activity. One bile ductule present shows ductulitis characterized by intraepithelial lymphocytes (blue arrow. Magnification, 400×. (B) Poorly formed granulomata (green arrows) are seen within the dense chronic inflammatory infiltrate involving a portal tract. Magnification, 400×. (C) The portal tract contains a bile duct demonstrating loss of the normal architecture as well as hypereosinophilia of the cytoplasm and focal cytoplasmic vacuolization, evidence of bile duct damage (orange arrow). Magnification, 400×.

Given the persistently elevated transaminases, liver biopsy was performed. The liver biopsy (Figure 1) showed lymphocytic ductulitis and bile duct damage with poorly formed granulomata, favoring the diagnosis of PBC. There was significant plasma cell infiltrate noted but with minimal interface activity, not meeting criteria for autoimmune hepatitis/overlap syndrome.

The markedly elevated AMA, associated with the characteristic histological findings of the liver biopsy, made the diagnosis of PBC the top differential diagnosis, without the need to pursue further biliary imaging. Additionally, there were no histologic features suggestive of small‐duct primary sclerosing cholangitis, including periductal fibrosis, fibro‐obliterative duct lesions, or chronic cholestatic changes in periportal hepatocytes. Sarcoidosis was considered in the context of hepatic granuloma; however, the presence of a normal chest X‐ray and predominant ductal inflammation on biopsy made this diagnosis unlikely. Immune‐mediated cholangiopathies were also considered but deemed unlikely due to the absence of biochemical cholestasis on laboratory monitoring.

**Figure 2:**
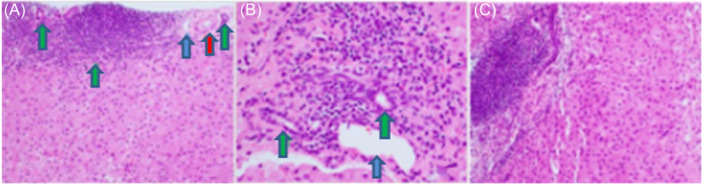
(A) Repeat biopsy: Despite the presence of chronic inflammation, the bile ducts are preserved and show no minimal or no evidence of bile duct damage (green arrows). The arteries and veins are not involved in the inflammatory process (red and blue arrows, respectively). Magnification, 400×. (B) Repeat biopsy: The chronic portal inflammation is characterized by a predominance of lymphocytes, occasional eosinophils, and rare plasma cells. The bile ducts are preserved and show no evidence of ductulitis or bile duct damage (green arrows). The vein is not involved by the inflammatory process (blue arrow). Magnification, 400×. (C) Repeat biopsy: Subset of portal tracts with persistence of the mild chronic portal inflammation without definitive interface activity and no granulomata. Magnification, 400×.

**Figure 3:**
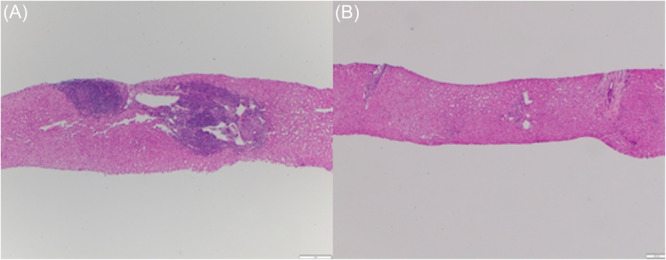
(A) The initial biopsy at diagnosis shows dense portal inflammation obscuring the components of the portal tracts. There is a predominance of lymphoid cells with numerous plasma cells. Magnification, 40×. (B) Note the marked response to treatment with a marked reduction in the chronic inflammation that was present in the portal tracts at the time of diagnosis. Magnification, 40×.

The patient was started on ursodeoxycholic acid (UDCA) at a dose of 300 mg twice daily with good response to the treatment. Repeat biopsy (Figure 2) 6 months later showed minimal ductal damage and mild portal lymphocytic infiltrate without interface activity, correlating with the normalized liver enzymes. Figure 3 shows a comparison of pre‐ and post‐treatment biopsies demonstrating marked improvement in inflammation.

## 3 DISCUSSION

PBC is a slowly progressive cholestatic liver disease occurring in genetically susceptible individuals. Exposure to an autoimmune triggering event can activate the immune response, leading to bile duct damage. Female gender and family history of PBC/other autoimmune conditions are known risk factors. During the early stages of the disease, patients are usually asymptomatic; some patients gradually develop symptoms of fatigue, pruritus, and jaundice. PBC is a rare condition in children, with only a few pediatric cases reported to date.^2^, ^3^, ^4^, ^5^ Age ranged between 5‐ and 12‐year‐old females with varying outcomes. Liver transplantation was reported for advanced liver disease with ultimate liver failure.

The adult guidelines require two of following criteria to confirm the diagnosis of PBC: (1) laboratory evidence of cholestasis with elevated Alk Phos, (2) positive AMA or other PBC specific antibodies including sp100 or gp120 if AMA is negative, and (3) histological confirmation with non‐suppurative cholangitis and destruction of small or medium sized bile ducts.^6^ Our patient did not meet laboratory criteria but did have a positive AMA and histological findings consistent with PBC. Measuring AMA is not routine practice in the evaluation of pediatric patients with cholestasis or elevated transaminases. While the positive AMA prior to the biopsy was of interest, we would have proceeded with biopsy regardless given the patient's history of elevated transaminases of unclear etiology.

UDCA is the first‐line treatment for PBC. It has substantially improved outcomes, altering the natural history of the disease, dividing into the pre‐UDCA and UDCA era. Studies have demonstrated that it not only improves biochemical markers but also histological markers, while reducing the rate of liver transplant.^7^ UDCA has multiple mechanisms, including choleretic, cytoprotective, anti‐inflammatory, and immunomodulatory properties.^8^ It protects cholangiocytes from bile‐induced oxidative damage by reducing hydrophobic bile acids, helps with stimulation of impaired hepatobiliary secretion of bile acids, and inhibits apoptosis of liver cells. UDCA is most effective when initiated early, but studies have shown benefits at any stage. Second‐line treatment agents are available if UDCA fails. If the patient develops cirrhosis, they may ultimately require a liver transplant.

## 4 CONCLUSION

Our case raises awareness of the possibility of PBC in children and highlights the importance of including AMA in pediatric patients presenting with elevated liver enzymes who otherwise have inconclusive laboratory findings. Currently, there is limited data on pediatric onset of PBC and its natural history, with only a few reports available. There are no established pediatric guidelines for the diagnosis of PBC; therefore, the use of adult diagnostic criteria represents a limitation when applied to pediatric patients. This case highlights gaps in the existing literature due to the limited availability of pediatric data.

## CONFLICT OF INTEREST STATEMENT

The authors declare no conflict of interest.

## ETHICS STATEMENT

Written informed patient consent was obtained from the parent.
